# Opportunities for Antigen Discovery in Metastatic Breast Cancer

**DOI:** 10.3389/fimmu.2020.570049

**Published:** 2020-10-30

**Authors:** Ashwani K. Sood, Michael Nemeth, Jianmin Wang, Yun Wu, Shipra Gandhi

**Affiliations:** ^1^ Department of Immunology, Roswell Park Comprehensive Cancer Center, Buffalo, NY, United States; ^2^ Department of Medicine, Roswell Park Comprehensive Cancer Center, Buffalo, NY, United States; ^3^ Department of Biostatistics and Bioinformatics, Roswell Park Comprehensive Cancer Center, Buffalo, NY, United States; ^4^ Department of Biomedical Engineering, University at Buffalo, The State University of New York, Buffalo, NY, United States

**Keywords:** CDK4/6 inhibition, *in situ* anti-tumor immunity, synergizing TAA and TNA, increased tumor immunity, decreased autoimmunity

## Abstract

Immune checkpoint inhibitor-based immunotherapy (ICI) of breast cancer is currently efficacious in a fraction of triple negative breast cancers (TNBC) as these cancers generally carry high tumor mutation burden (TMB) and show increased tumor infiltration by CD8^+^ T cells. However, most estrogen receptor positive breast cancers (ERBC) have low TMB and/or are infiltrated with immunosuppressive regulatory T cells (Tregs) and thus fail to induce a significant anti-tumor immune response. Our understanding of the immune underpinning of the anti-tumor effects of CDK4/6 inhibitor (CDKi) treatment coupled with new knowledge about the mechanisms of tolerance to self-antigens suggests a way forward, specifically *via* characterizing and exploiting the repertoire of tumor antigens expressed by metastatic ERBC. These treatment-associated tumor antigens (TATA) may include the conventional tumor neoantigens (TNA) encoded by single nucleotide mutations, TNA encoded by tumor specific aberrant RNA transcription, splicing and DNA replication induced frameshift (FS) events as well as the shared tumor antigens. The latter may include the conventional tumor associated antigens (TAA), cancer-testis antigens (CTA) and antigens encoded by the endogenous retroviral (ERV) like sequences and repetitive DNA sequences induced by ET and CDKi treatment. An approach to identifying these antigens is outlined as this will support the development of a multi-antigen-based immunotherapy strategy for improved targeting of metastatic disease with potential for minimal autoimmune toxicity against normal tissues.

## Introduction

ICI has ushered in a revolution in the treatment of cancer, especially of advanced metastatic disease where other treatments have shown limited success (1). However, only a few cancer types including melanoma, lung cancer, bladder cancer, kidney cancer, liver cancer, head and neck cancer as well as certain cancers with high microsatellite instability are responsive to ICI. A distinguishing characteristic of these cancers is that they carry high TMB (2, 3) reviewed in (4) that results in the expression of increased numbers of immunogenic TNA. High predicted TNA number was correlated with increased tumor infiltration of CD8^+^ T cells and increased survival of cancer patients (5). Additionally, the number of predicted MHC class I associated TNA showed positive correlation with intra tumoral level of transcripts associated with NK and T cell mediated cytotoxic activity across various cancer types (6). Together, these and other studies suggested that the anti-tumor immune response is primarily focused on TNA [reviewed in (7, 8)].

However, even within the ICI responsive cancer types, only a minority of patients (20–40%) may experience significant tumor shrinkage and/or prolonged cancer remission. This presents a challenge of how to bring the benefits of immunotherapy to majority of the patients with TNA-high cancer types. Another significant challenge facing the cancer immunotherapy community is how to advance the application of immunotherapy to cancer types that are typically poorly responsive to ICI. These include cancers of the breast, prostate, ovary, pancreas, glioblastoma and other cancers of the brain and several hematopoietic malignancies. These cancers are characterized generally by low TMB, expression of fewer TNA, and poor infiltration by T cells (2–4). These cancers are also known as immunologically inert or “cold tumors”. How to convert these TNA-low cold tumors into immunologically active T cell infiltrated “hot tumors” remains a largely unsolved problem in the contemporary cancer immunotherapy research.

According to American Cancer Society, the 5-year survival rate for metastatic breast cancer is <30%. This represents a major departure from earlier stages of breast cancer for which there are impressive 5-year survival rates ranging from 72% for stage III, >93% for stage II and 100% for stage I breast cancer. Nevertheless, the grim outlook for metastatic disease may improve due to the recent advances in the application of immunotherapy to a fraction (about 40% of tumors with PD-L1 expressing immune infiltrate) of TNBC, resulting in significantly improved progression free and overall survival (9, 10). TNBC carry higher TMB compared to ERBC (11, 12). Also, patients with TNBC and Her2+ breast cancers (Her2BC), especially those with high TMB, showed a linear relationship between increased numbers of tumor infiltrating T cells and improved recurrence-free and overall patient survival (13–15). In the neoadjuvant setting as well, increased lymphocytic infiltration showed significant association with pathologic complete response (pCR) to chemotherapy (16, 17). However, difference in pCR was primarily significant for TNBC and Her2BC and was not observed for ERBC (17). These findings are consistent with the improved clinical outcomes observed following ICI of TNBC (9, 10).

In contrast, excepting a small fraction of ERBC with high mutation load, immune infiltration and improved survival (15), very few studies have documented significant CD8^+^ T cell infiltration in most ERBC which comprise more than two thirds of all newly diagnosed breast cancers. This may be due to: i) the lower tumor mutation load in ERBC and/or ii) the specific biology of this tumor subtype since independent studies showed increased infiltration of immunosuppressive T regulatory cells (Tregs) in this breast tumor subtype (18, 19). Hence, although metastatic ERBC may show a higher number of nonsynonymous mutations (>2-fold higher compared to primary tumors) (20), the presence of immunosuppressive Tregs may pose a significant barrier to the induction and function of anti-tumor T cells against metastatic disease.

## Advances in Targeted Therapy of Metastatic ERBC Suggest a Way Forward

Advances in the targeted therapy of metastatic ERBC with ET plus CDKi appears to offer new hopes for expanding the application of immunotherapy to the treatment of ERBC. Briefly, a recently published phase III trial (MONALESSA-7) tested CDKi (ribociclib) in combination with endocrine therapy for premenopausal or perimenopausal women with locally advanced and metastatic ERBC. The results showed an overall survival rate of 70.2% for endocrine therapy plus ribociclib group compared to 46% for endocrine therapy alone group, after 42 months of follow-up period (21). This new treatment modality is expected to show significant improvement in the 5-year survival rates, should this patient group continue to do well for a longer follow up period. Additionally, progression free and overall survival in this study was similar to other Phase III studies in postmenopausal women using other CDKi (abemaciclib or palbociclib) plus endocrine therapy (22–24). These clinical outcomes are impressive and provide a new benchmark for future improvements in the treatment of metastatic breast cancer.

Pre-clinical studies have been conducted to understand the underlying mechanisms (25–27). Collectively, these studies showed that CDKi treatment have multiple effects on the tumor and tumor microenvironment as well as systemic effects on immune cells. Specifically, in tumor cells, CDKi induces tumor cell stasis and down regulates DNA methyl transferase 1 (DNMT1) levels. This leads to hypomethylation-mediated induction of endogenous retroviral sequences, Type III IFN production and increased expression of IFN signaling related molecules and of interferon stimulated genes including genes involved in antigen processing and presentation (25). As a result, the expression of MHC class I (MHC-I) antigens is increased on the surface of tumor cells rendering these cells susceptible to increased recognition and killing by cytotoxic T cells. Further, CDKi enhances the nuclear retention, hence, transcriptional activity of NFAT (nuclear factor of activated T cells) resulting in increased expression of IL2 and IFN-γ, leading to increased proliferation and anti-tumor function of activated T cells (26). Increased expression of type I chemokines CXCL9 and CXCL10 by activated dendritic cells was also observed in the tumor microenvironment. This is expected to promote increased tumor infiltration of activated T cells and render the tumor microenvironment more TH1-like and immune stimulating (25–27). Systemically, reduced numbers of Tregs were seen in the spleen and lymph node of mice subjected to CDKi, without signs of autoimmunity (25). On the other hand, CD8^+^ T cell numbers were slightly reduced while the numbers of conventional CD4^+^ T cells remained unchanged (25). Thus, by decreasing Treg numbers systemically, CDK4/6 therapy likely supports increased priming of tumor-antigen specific T cells in tumor draining lymph nodes and their subsequent expansion in the tumor microenvironment (25–27). However, the nature of the tumor antigens recognized in this process remains unknown.

As noted previously (7, 8), a possibility is that CDKi induced tumor immunity primarily targets TNA. These may include the conventional TNA arising from single nucleotide changes. Other TNA may arise from frame shift mutations resulting from errors of RNA transcription and splicing (28, 29) as well as novel protein coding sequences resulting from intron retention (30). DNA replication of microsatellites may also generate TNA (31). These antigens are likely to be specific to individual patients as they result from stochastic events. The combination of ET plus CDKi therapy may generate additional TNA through induction of promoter hypomethylation and subsequent expression of endogenous retroviral like sequences (ERV) and transposable elements (32). Also, hypomethylation induced use of cryptic transcription start sites (TSS) may occur, as was previously reported by studies that used other DNA methyl transferase inhibitors (DNMTi) and histone deacetylase inhibitors (HDACi) (33). In this study, cryptic TSS were primarily found in long terminal repeats of the LTR12 family and may encode 5 ´truncated proteins as well as proteins arising from novel splicing of altered 5 ´transcripts (33). As these proteins may be shared between individuals, they are a potential source of shared TNA.

Besides TNA, it is plausible that the CDKi induced immune stimulating conditions are conducive to priming T cell responses against non-mutated tumor associated antigens (TAA) including cancer-testis antigens (CTA) that are induced during ET plus CDKi treatment of metastatic disease. Many examples of breast TAA exist including human epidermal growth factor receptor 2 (Her2), Carcinoembryonic antigen (CEA), Mucin 1 (MUC1), Mammaglobin A (SCGB2A2), Alpha Lactalbumin (LALBA), Cyclin D1 (CCND1), Folate receptor 1 (FOLR1), Wilms tumor 1 (WT1), Survivin (BIRC5) and Telomerase (TERT) (reviewed in (34, 35)). However, many of these antigens may not be available following treatment with ET plus CDKi due to the loss of or blocking of estrogen signaling by ET and arrest of cell cycle progression by CDKi. Thus, antigens normally induced by estradiol or those induced during cell cycle progression may not be available on tumors during and/or following ET plus CDKi treatment. On the other hand, antigens whose expression is negatively regulated by estradiol or by E2F transcription factors may become induced during ET plus CDKi treatment and become available for targeting. One example of how endocrine therapy may modulate tumor antigen expression comes from an observation that estradiol negatively regulates Prostate-derived Ets factor (PDEF) expression (36), which is a candidate breast tumor antigen (37, 38). The loss of estradiol signaling during ET should therefore lead to increased PDEF expression in tumor cells resistant to ET (39, 40). Similarly, CTA expression is particularly sensitive to control by DNA methylation and the loss of DNMT1 during CDKi treatment will likely modulate the expression of many CTA that are expressed in breast cancer (41, 42). A prominent example of how epigenetic modulators induce CTA expression comes from studies in multiple tumor types demonstrating that these drugs remove epigenetic silencing of promoters associated with CTA genes (43–45). This may directly cause the expression of immunogenic TAA that can serve as targets of spontaneous or vaccine-induced anti-tumor immunity. These considerations underscore the importance of characterizing the antigenic profiles of ET plus CDKi treatment resistant metastatic tumor lesions as they are likely to be different from the antigenic profiles of the treatment naïve primary tumors.

## New Understanding of Mechanisms of Tolerance Supports TAA as Targets of CDKi Induced Anti-Tumor Immunity

TAA have been the basis of therapeutic vaccines against cancer over the past several decades. However objective responses in clinical trials, especially in the setting of advanced disease in solid tumors have been limited. This brought into question the continuing development of TAA based therapeutic vaccines for cancer immunotherapy (46). It was hypothesized that due to the mechanisms of tolerance, TAA specific high avidity T cells are deleted in the thymus. The remaining low avidity T cell responses to TAA are ineffective in reducing tumor burden, especially in the immunosuppressive microenvironment of advanced cancers. To the contrary however, recent observations suggest that TAA specific T cells are present in the periphery and have the potential to show anti-tumor effects. Specifically, studies on the mechanisms of tolerance to self-antigens showed incomplete deletion of self-antigen reactive T cells in the thymus. Thus, the frequency of naïve CD8^+^ T cells reactive to epitopes from self-antigens was found to be similar to that seen for T cell epitopes from viral proteins (47). Also, 30% of T cells reactive to male antigen H-Y were retained in male mice compared to female mice, showing incomplete deletion of self-antigen reactive T cells in male mice (47). Moreover, the affinity of the H-Y specific T cells in male and female individuals was comparable although the H-Y specific T cells from males were refractory to antigen-dependent stimulation suggesting some form of anergy (47). Similar to CD8^+^ T cells, CD4^+^ T cells specific to bacteriophage Cre protein expressed as a self- antigen in a tissue-restricted fashion were not deleted during their maturation in the thymus. Rather tolerance to this tissue restricted self-antigen was mediated primarily by Tregs (48). Significant deletion of Cre specific CD4^+^ T cells was only observed when this self-antigen was expressed ubiquitously. Thus, this current work (47, 48) showed a limited role for thymic deletion as a mechanism of tolerance contrary to the prior work with T cell receptor transgenic mice that showed thymic deletion of self-antigen reactive T cells as a robust mechanism of tolerance to self-antigens. In the current work, major mechanism of tolerance to self-antigens appears to be mediated by antigen specific Tregs (48) that suppress the proliferation and/or activation of self-reactive T cells (47, 48). These findings are consistent with other seminal observations that showed that impaired development of Tregs in Foxp3 mutant mice or depletion of Foxp3^+^CD25^+^ Tregs results in widespread multi-organ autoimmunity (49, 50). Together, these studies support the notion that self-tissue antigen or TAA specific T cells with potential for auto-immune pathogenicity/anti-tumor activity are present in the periphery but kept in check by Tregs. Moreover, Treg mediated tolerance appears to be unstable and was abrogated by repeated immunization with Cre antigen (48) and other self-antigens (51). This observation and the immune underpinning of CDKi treatment suggest that CDKi mediated induction of tumor cell stasis, reduction in Treg numbers and induction of TH1 type immune stimulating tumor microenvironment together may tip the balance from Treg mediated immune suppression towards priming of endogenous T cell responses to TAA expressed by ERBC.

There are several advantages of using TAA in therapeutic vaccination against cancer. These include: i) TAA provide off the shelf peptide, DNA or RNA reagents for use in immunizations since they are expressed frequently in significant fractions of tumors; ii) Patients may be screened for pre-existing immunity to specific TAA so that patient specific immunogenic TAA may be selected for immunization of individual patients to boost their anti-tumor immunity. This is an important advantage of TAA particularly in the setting of metastatic disease since metastatic lesions may not be easily accessible for identification of TNA or TAA with the exception of screening a peptide library of predicted FS peptides and/or TAA peptides to identify immunogenic FS TNA (28) or TAA; iii) Most TAA are products of oncogenes that are required for tumor growth/survival hence their loss by tumor cells to escape TAA specific tumor immunity is less likely (34). In contrast, only 8% of TNA are derived from cancer driver genes, the vast majority (92%) are derived from passenger genes that are not required for tumor growth/survival (4). The latter TNA are prone to be lost from tumor cells and thus promote resistance to immunotherapy (52, 53); iv) Each TAA may contain several immunogenic epitopes in the context of multiple HLA-alleles; hence induction of immune response against individual TAA may be feasible in most patients (54, 55). Specifically, two groups have independently tested NY-ESO-1–specific long peptides as vaccines in ovarian and melanoma cancer patients and found that in the presence of appropriate adjuvants most patients responded with integrated antibody and CD4^+^ T and CD8^+^ T cell responses against NYESO-1, irrespective of patients HLA type (54, 55). Apparently, multiple NY-ESO-1 peptides in the context of polymorphic HLA alleles are the targets of T cells in these studies. On the other hand, most breast cancers may only have a few TNA (11, 12) and their immunogenicity is further limited by the frequency of the HLA-allele to which they bind.

Importantly, metastases may differ in their antigenic profile from primary tumors due to genetic evolution (56–58) and, as mentioned previously, due to the ET plus CDKi treatment induced changes in gene expression in metastatic lesions. A recent study further showed that nearly half of the significantly mutated genes in metastases were previously not described as significantly mutated in primary breast tumors (59). These findings coupled with preferential changes in several oncogenic pathways suggest that metastases are biologically distinct from primary breast tumors (59). However, the degree of diversity between metastases from different patients may not be ascertained from this study since patients were subjected to different treatment regimens including, radiation therapy, chemotherapy, endocrine therapy and anti-Her2 therapy. On the other hand, when treated uniformly with ET and CDKi, metastases may exhibit lower diversity, such that different metastases within and across patients may express shared tumor antigens. From these considerations, it seems that characterization of both TNA and TAA that are expressed in ET plus CDKi treatment resistant metastases and are immunogenic in individual breast cancer patients (undergoing ET plus CDKi treatment) is desirable to developing a multi-antigen-based vaccine approach for immunotherapy of metastatic disease.

## TNA and TAA May Synergize to Induce Robust Tumor Immunity

As mentioned previously, the presence of high TMB coupled with CD8^+^ T cell infiltration show association with improved patient survival (13–17). This implied the recognition of MHC-I binding TNA as targets of protective CD8^+^ T cell immunity against breast cancer. This idea has received validation in studies that used single nucleotide derived nonsynonymous TNA based vaccines or TNA specific ex vivo amplified TILs in adoptive therapy of breast cancer (60, 61). Other studies in future would likely validate other forms of TNA (28–33) that may be expressed by metastatic ERBC and serve as targets of anti-tumor CD8^+^ T cell immunity. While these studies (60, 61) are pioneering, their general applicability is dependent on the availability of TNA-derived MHC-I binding CD8^+^ T cell epitopes. This however may be challenging. Specifically, besides being potentially rare in ERBC, most MHC-I binding TNA appear to induce CD4^+^ T cell responses rather than the expected CD8^+^ T cell responses. Thus, studies with melanoma TNA vaccines showed that majority (60% or more) of the TNA predicted to bind to MHC-I antigens elicited CD4+ T cell responses (62, 63). Additionally, recent studies with glioma patients tested MHC-I binding TNA alone or mixtures of MHC-I binding TNA and TAA for immunization and found that TNA alone vaccines mostly induced CD4^+^ T cell responses. In contrast, vaccines containing both TNA and TAA epitopes or those containing only TAA epitopes induced robust CD8^+^ T cell responses (64, 65). These studies suggest that inclusion of MHC-I binding epitopes from TAA in vaccine formulations to provide CD8^+^T cell epitopes may be critical to inducing robust CD8^+^ T cell responses against metastatic breast cancer (64–66).

Whereas the importance of MHC-I restricted CD8^+^T cell responses to anti-tumor immunity is undisputed, it is also well established that CD8^+^ T cell responses themselves are critically dependent on the availability of CD4^+^ T cell help [(67–71), reviewed in (72)]. This help is provided in the form of secreted cytokines by CD4^+^ T cells and *via* CD40 ligand mediated signaling to antigen presenting cells (APC) for activating the CD70 mediated costimulatory functions of APC for CD8^+^ T cell clonal expansion and for the development of cytotoxic function and of memory phenotype of CD8^+^T cells. Moreover, CD4^+^ T cell mediated help appears to be most effective when both helper and cytotoxic T cell epitopes are presented on the same APC (72). In this regard, the putative MHC-I binding TNA that induce CD4^+^ T cell responses (presumably *via* binding to Class II MHC (MHC-II) molecules) together with MHC-II binding TNA are useful helper epitopes as they are expressed by the same tumor cells as the TAA and therefore captured together by APC for cross presentation to both CD4^+^ and CD8^+^ T cells (72). Together these observations provide a rationale for characterizing both TNA and TAA expressed by metastatic breast tumor cells for inducing robust anti-tumor immunity in most breast cancer patients.

## CDKi Treatment While Improving Anti-Tumor Immunity May Limit Autoimmune Toxicity Against TAA Expressing Normal Tissues

The potential to induce TAA specific strong T cell responses against ERBC also raises concerns about the risk of autoimmune toxicity against TAA expressing normal tissues. Specifically, in clinical studies strong anti-tumor effects of TAA specific adoptively transferred T cells correlated with autoimmune pathogenicity against TAA expressing normal tissues (reviewed in (73)). These studies however used a pre-conditioning regimen of chemotherapy with cyclophosphamide. Cyclophosphamide treatment is known to deplete Tregs (74, 75) and this may have promoted increased autoimmune toxicity of anti-tumor T cells against TAA expressing normal tissues. These findings emphasized the need to selectively augment TAA directed anti-tumor immunity, while sparing the destruction of TAA expressing normal tissue(s). To that end, it seems that a degree of specificity for increased targeting of tumor cells compared to TAA-expressing normal tissues may be achieved by CDKi treatment. by preferentially targeting rapidly dividing tumor cells, CDKi treatment may increase TAA expression and its processing and presentation in complex with MHC-I on tumor cell surface (25). This renders tumor cells more susceptible to killing by TAA specific cytotoxic T cells. Also, the TH1 type immune stimulating tumor microenvironment induced by CDKi treatment and its promotion of increased infiltration of activated T cells into the tumor tissue together could promote increased intra-tumor T cell expansion and anti-tumor effects (26). In contrast, these CDKi-mediated effects may not occur in non-dividing normal tissues, which may use an alternative cell cycle signaling pathway involving cyclin E-CDK2 with a prolonged G1 phase (76). This is consistent with the limited side effects with CDK4/6 inhibition observed against normal tissues. CDKi treatment therefore has the potential to preferentially support the immunotherapy of tumor tissue. Some systemic depletion of T regs also occurs in CDKi treated mice (25), but this was not sufficient to elicit an autoimmune phenotype in these mice. Since CDKi treatment is now a part of the standard treatment for metastatic ERBC, all metastatic tumor lesions in a patient may be preferentially susceptible to TAA targeted immunotherapy with limited autoimmune toxicity against normal tissues. Further increase in the specificity of tumor immunotherapy may occur when targeting multiple TAA such that an individual normal tissue by expressing a limited subset of the TAA may experience further limited autoimmune toxicity.

## Discussion

**Figure 1:**
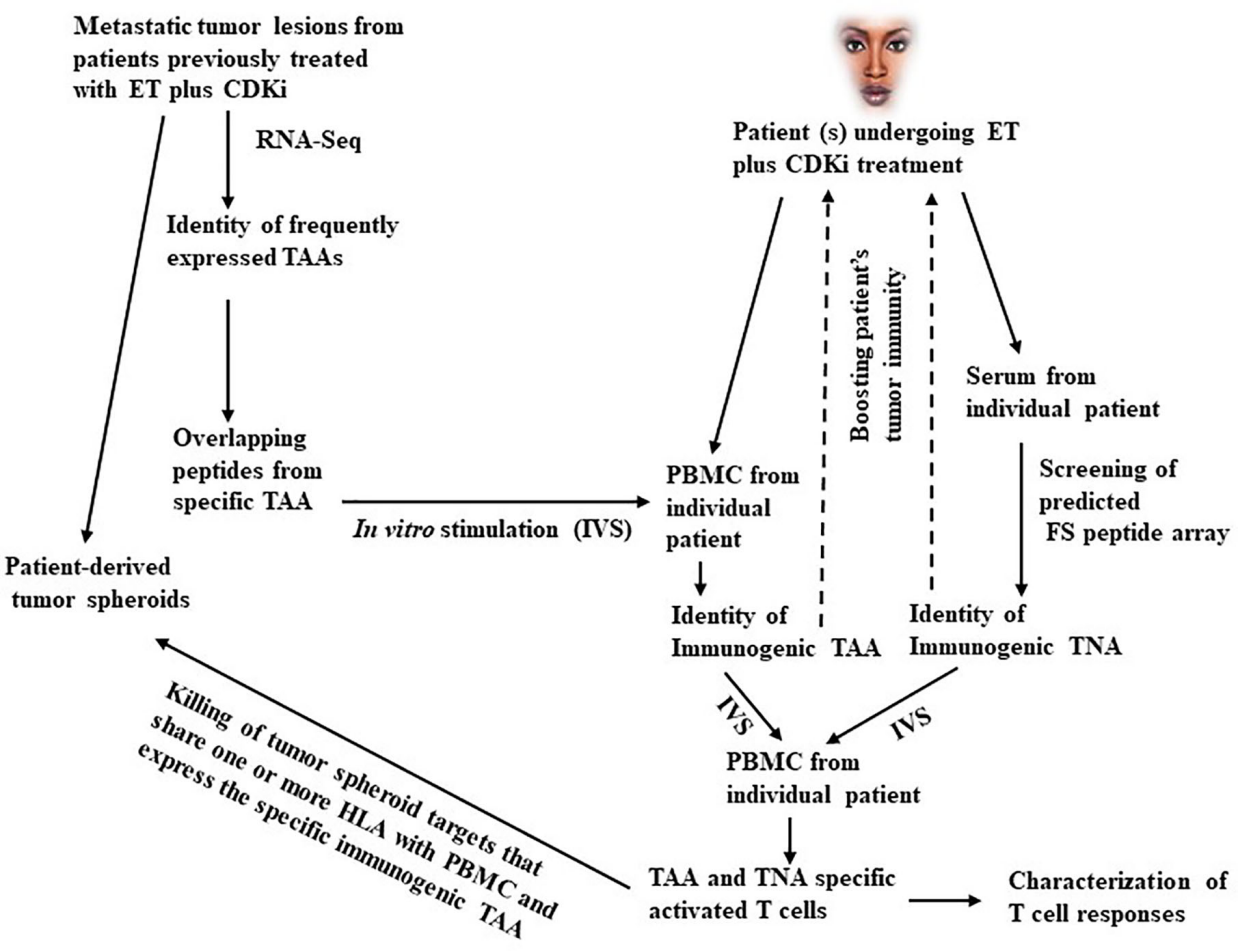
A schematic outline of the antigen discovery approach in metastatic breast cancer. As shown in the left part of this figure, metastatic tumor lesions arising in patients treated with ET plus CDKi are obtained. A part of the tumor is used for developing tumor spheroid cultures. The remaining tumor is used for RNA-Seq and CAGE-Seq analysis to identify the frequently expressed TAA. Based on the individual TAA sequence, overlapping peptides are synthesized and used to pulse DC to *in vitro* stimulate PBMC from individual patients undergoing ET plus CDKi treatment to identify immunogenic TAA. Simultaneously, patient’s serum is used to screen a FS peptide array to identify immunogenic TNA. The mixture of immunogenic TAA and TNA is further tested for ability to stimulate and expand TAA specific T cell responses *in vitro* with capacity to kill tumor spheroid targets that share one or more HLA class I antigens with patient’s T cells and that also express the specific immunogenic TAA. The use of tumor spheroids is important here since they represent the metastatic tumor lesions that arose following treatment with ET and CDKi. Hence, they are likely to exhibit the treatment induced gene expression and antigenic profile as well as the changes associated with the development of treatment resistance. Moreover, the tumor spheroid may express natural levels of antigen overexpression, they are therefore more suitable targets for testing the anti-tumor activity of TAA and TNA specific cytotoxic T cells. On the other hand, tumor cell lines may be less suitable targets since they may lack the antigenic profile characteristic of the exposure to ET plus CDKi treatment. Moreover, following transfection with antigen encoding gene the transfectant tumor cell lines may express unnaturally high levels of the tumor antigen and be more susceptible to cytotoxic T cell killing compared to tumor spheroids. Nevertheless, transfectant tumor cell lines may be a useful alternative to tumor spheroids in the event the relevant tumor spheroid targets are unavailable. The validated TAA and TNA are then tested for their safety and capacity to boost patient’s anti-tumor immunity (as indicated by dotted arrows).

Whereas a fraction of TNBC with relatively high mutation load and T cell infiltration are responsive to ICI, most ERBC have low tumor mutation load that appears inadequate for priming a conventional TNA targeted CD8^+^ T cell response with anti-tumor efficacy. The immune underpinning of the anti-tumor effects of CDKi treatment therefore provides an exciting new opportunity to identify additional TNA and TAA induced and recognized by this treatment induced immunity. Specifically, candidate TNA and TAA may be predicted from exome sequencing, CAGE-seq, and RNA-seq (29–33) of biopsies from patient’s tumor lesions that progress on ET plus CDKi treatment. This is followed by testing the candidate antigens as targets of CDKi induced anti-tumor T cell immunity. Also, patient’s serum may be used to screen a library of predicted FS peptides (28) to identify immunogenic FS TNA. The knowledge of the immunogenic TAA and TNA will support the development of multi-antigen vaccines to boost the CDKi treatment induced immunity to prolong disease remission and improve survival of patients from metastatic breast cancer (a schematic of this process is shown in **Figure 1**). In the future, this approach may also be applicable to preventing cancer recurrence in early stage ERBC patients treated with adjuvant ET plus CDKi (if approval obtained in the adjuvant setting as these trials are currently underway). Specifically, following ET plus CDKi treatment, patients may be monitored for disease progression *via* circulating tumor DNA (ctDNA). Results show that following primary treatment, disease progression can be detected by monitoring ctDNA levels by a median of ~10 months in advance of the clinical detection (77, 78). This provides a window of opportunity to determine the immunogenic antigens targeted by ET plus CDKi induced tumor immunity in individual patient, allowing immunotherapy in the setting of minimal residual disease. If demonstrated to be useful in breast cancer, this approach will have application in other cancers as well that are responsive to CDKi treatment. Pre-clinical studies in cell culture and mouse models and early phase clinical trials support the potential of CDKi for treatment of other cancers including bladder cancer (79), endometrial cancer (80), glioblastoma (81), multiple myeloma (82), mantle cell lymphoma (83), Ewing sarcoma (84) and potentially other cancers (reviewed in ref. (85)).

The following discussion specifically focuses on the methodologic considerations and clarifications. One consideration pertains to the specific properties of a TAA that render it potentially immunogenic. To that end, expression level of TAA is an important determinant of its immunogenicity. Equally important, however, is the affinity of the TAA derived peptide epitope(s) for binding to class I MHC (MHC-I) molecules. Hence it is difficult to assign a specific value for the level of TAA or TNA expression alone as a prerequisite for considering a TAA to be potentially immunogenic. Moreover, to serve as a target of cytotoxic T cell mediated tumor cell killing only a few TAA-derived peptide -MHC complexes may be needed on the tumor cell surface (86). Additionally, the number of TAA peptide-MHC complexes on tumor cell surface may be influenced by the tumor microenvironment, a Type I tumor microenvironment is conducive to increased expression of MHC-I molecules, hence of peptide-MHC complexes (25). This suggests that a variable level of TAA expression may be adequate for efficient presentation of different TAA in specific tumor microenvironments. To ensure that all potentially immunogenic TAA are evaluated, a >2-fold higher level of TAA expression in the tumor tissue compared to the benign breast tissue may be considered a minimum threshold overexpression for testing a TAA for potential immunogenicity. To facilitate the identification of overexpressed TAA, a mixture of RNA from benign breast tissues may be included in the RNA-seq analysis for comparison with the tumor tissue. The immunogenic FS TNA on the other hand are identified by reactivity with patient’s serum following screening of the predicted FS peptide array (28).

Mutations in driver oncogenes deserve additional considerations as they can be used both as TNA and/or TAA. Specifically, mutant KRAS is a driver oncogene in pancreatic, lung and colon cancers and in a small fraction of breast tumors. The RasG12D mutations was successfully targeted by specific TCR based adoptive immunotherapy (87). Because of the tumor specificity of the mutation there was little concern that adoptively transferred T cells may trigger autoimmune toxicity against normal tissues. Additionally, mutant KRAS TNA epitopes as well as certain wild type KRAS epitopes were shown to be useful as a vaccine for inducing specific T cell responses (88). Similarly, while certain TP53 mutations including frame shift and nonsense mutations result in the loss of TP53 expression and function, other missense mutations result in stable expression of mutant TP53 and may function as dominant negative oncogenes that may suppress the wild type p53 function (89). Moreover, these TP53 mutants may show overexpression in the tumor tissue compared to normal tissues since wild type TP53 in normal tissues is targeted for degradation. Therefore, the mutant TP53 may serve as a TNA (due to its tumor specificity) and a TAA due to its potential oncogenicity and overexpression in the tumor tissue and weak expression in normal tissues.

In **Figure 1**, overlapping long peptides from TAA are used for *in vitro* stimulation of *in situ* primed PBMCs from patients undergoing ET plus CDKi treatments for identification of immunogenic TAA. This is an unbiased approach for identifying immunogenic peptides in the context of all HLA alleles. In contrast, the alternative MHC-I binding peptide prediction approach may not be optimally predictive of potentially immunogenic peptides especially in the context of less frequent HLA alleles. The latter peptides may therefore be missed by MHC-I binding peptide prediction approach. Moreover, other approaches that use TILs screening may not be viable as TILs are poorly available from most patients undergoing ET plus CDKi treatment due to the poor availability of metastatic tumor lesions. The goal of our approach is to inhibit metastatic tumor progression by augmenting patient’s anti-tumor immunity. To that end, our use of IVS assay to identify the immunogenic TAA is novel since it is based on the expectation that PBMCs from ET plus CDKi treated patients are likely to contain *in situ* primed TAA specific T cells. Hence this IVS assay should be more sensitive and should facilitate the identification of immunogenic TAA and TNA.

Additionally, in **Figure 1**, the suggested treatment approach could be vaccination, TCR-T cell adoptive transfer or both. Our preference is for vaccination, especially when targeting a TAA since it is relatively safer compared to adoptive T cell transfer. The latter requires lymphodepletion that also depletes Tregs and may render normal tissues expressing the TAA susceptible to auto-immune toxicity by adoptively transferred T cells. However, TNA from oncogenes like KRAS, ER or mutant TP53 will be ideal targets of both vaccination and adoptive T cell therapy since they are tumor specific, obviating any concerns for autoimmunity against normal tissues.

It is well known that patients with luminal B breast cancer have a higher proliferation index and higher TIL-levels, than patients with luminal A cancer. This raises the questions whether luminal B patients may be more responsive to antigen-directed treatments than luminal A patients? An answer to this question may depend on whether the metastases from luminal B tumors (with higher T cell infiltrate) will remain more immunogenic compared to metastases from luminal A tumors (with lower T cell infiltrate). A preliminary answer may be derived from the levels of TAA specific T cell responses in the IVS assay. A higher T cell stimulation with PBMCs from patients with luminal B primary tumors compared to those with luminal A primary tumors will be consistent with increased immunogenicity of metastases arising in patients with luminal B tumors. Based on these observations, patients with luminal B tumors will be expected to be more responsive to antigen targeted vaccine/immunotherapies compared to luminal A patients. On the other hand, due to the considerable genetic evolution of the metastases and increased activation of metastases specific oncogenic pathways (56–59), coupled with exposure to ET plus CDKi treatment, metastases from both luminal A and luminal B tumors may become immunologically more similar, and these patients may show similar levels of TAA specific T cell responses in the IVS assay. In that event, luminal B and luminal A patients may respond similarly to antigen-directed vaccine-immunotherapies.

A related question is whether patients may be selected based on the TIL quantities in primary tumors irrespective of the tumor subtype (90) An answer to this question may again dependent on whether patients with higher TILs in primary tumors respond with higher levels of TAA-specific T-cell responses in the IVS assay compared to patients with low TILs in primary tumors. If so, then patients may be selected according to high TIL quantity (91). On the other hand, if patients with high or low TILs in primary tumors show similar levels of TAA specific T cell responses in the IVS assay due to similar immunogenicity of the metastases, this would preclude patient selection based on the quantitative T cell infiltration of primary tumors.

The expanded TILs from breast cancer patients with memory T cell phenotype were shown to determine good prognosis (92), suggesting that immune memory T cell phenotype of a T cell infiltrate is critical to the success of T cell therapies. This finding is consistent with other observations that show that an antigen specific mixed immune T cell response consisting of short-lived effector CD8^+^T cells and long-lived memory CD8^+^T cells is necessary for anti-tumor control (93, 94). Based on this understanding our inclusion of FS peptides to induce CD4^+^T helper cell responses is designed to promote both clonal expansion of TAA-specific effector CD8^+^T cells and their differentiation into central and effector memory phenotypes (67–72). Accordingly, following vaccination with TAA and TNA patients should be evaluated for the level and quality of T cell responses induced to determine whether there is an association between antigen specific T cell memory phenotypes and improved patient outcome.

Finally, we recognize that this is primarily a hypothesis generating perspective. However, the overarching approach developed in this article is novel and addresses an important unmet need, i.e., the immunotherapy of luminal breast cancer. Specifically, our approach is based on: i) novel understanding of the mechanisms of tolerance; ii) conceptual recognition of ET plus CDKi treatment induced antigens preferentially expressed in metastatic disease and iii) the potential for CDKi induced immune stimulating conditions to facilitate *in situ* priming of immune responses against these antigens. Together, this strategy appears realistic and could lead to the discovery of antigens preferentially expressed in metastatic disease and to the development of immunotherapeutic approaches for treatment of metastatic luminal breast cancer which has remained refractory to this treatment modality.

## Data Availability Statement

The original contributions presented in this study pertain to a novel approach to antigen discovery in metastatic breast cancer. Further enquiries can be directed to the corresponding author.

## Author Contributions

AS and SG conceived the idea, designed the manuscript outline and wrote an initial draft of the manuscript. MN, JW, and YW reviewed the relevant literature and provided critical insights that resulted in a substantially revised and improved manuscript. All authors contributed to the article and approved the submitted version.

## Funding

This work was supported by a grant from Breast Cancer Coalition of Rochester NY (to AS) and grants from National Center for Advancing Translational Sciences of the National Institute of Health under award number 5KL2TR0013-05 and UL1TR0012-05 (to SG).

## Conflict of Interest

The authors declare that the research was conducted in the absence of any commercial or financial relationships that could be construed as a potential conflict of interest.
